# A Retrospective Study of the Impact of the COVID-19 Pandemic on the Utilization and Quality of Antibiotic Use in a Tertiary Care Teaching Hospital in Low-Resource Settings

**DOI:** 10.3390/antibiotics14060535

**Published:** 2025-05-23

**Authors:** Vedrana Barišić, Tijana Kovačević, Maja Travar, Ana Golić Jelić, Pedja Kovačević, Dragana Milaković, Ranko Škrbić

**Affiliations:** 1 Clinical Pharmacy Department, University Clinical Centre of the Republic of Srpska, 78000 Banja Luka, Bosnia and Herzegovina or tijana.kovacevic@med.unibl.org (T.K.); dragana.milakovic@kc-bl.com (D.M.); 2 Faculty of Medicine, University of Banja Luka, 78000 Banja Luka, Bosnia and Herzegovina or maja.travar@kc-bl.com (M.T.); ana.golic@med.unibl.org (A.G.J.); pedja.kovacevic@med.unibl.org (P.K.); ranko.skrbic@med.unibl.org (R.Š.); 3 Department of Clinical Microbiology, University Clinical Centre of the Republic of Srpska, 78000 Banja Luka, Bosnia and Herzegovina; 4 Academy of Sciences and Arts of the Republic of Srpska, 78000 Banja Luka, Bosnia and Herzegovina; 5 Department of Pathologic Physiology, I.M. Sechenov First Moscow State Medical University, Moscow 119435, Russia

**Keywords:** antibiotic utilization, defined daily dose (DDD), COVID-19, AWaRe classification, tertiary care hospital

## Abstract

**Background/Objectives:** Improper use of systemic antibiotics remains a significant concern in hospital settings, contributing to increased antimicrobial resistance and suboptimal clinical outcomes. The COVID-19 pandemic exacerbated this issue. This study aimed to evaluate long-term trends in antibiotic utilization in low-resource settings at a tertiary care teaching hospital, focusing specifically on the changes before, during, and after the COVID-19 pandemic. **Methods:** This retrospective observational study analyzed antibiotic utilization data from the University Clinical Centre of the Republic of Srpska over ten years (2015–2024). Antibiotic consumption was expressed in defined daily doses (DDD) per 100 bed-days, and compared across three periods: pre-COVID-19 (2015–2019), COVID-19 (2020–2022), and post-COVID-19 (2023–2024). Additionally, antibiotic use was categorized according to the WHO AWaRe classification. **Results:** Antibiotic utilization peaked during the COVID-19 period, with the highest rate observed in 2021 (91.5 DDD/100 bed-days), despite a decrease in hospital admissions. The most frequently used antibiotics were cephalosporins, penicillins, and metronidazole. A significant increase in the use of azithromycin, meropenem, piperacillin/tazobactam, vancomycin, and colistin was noted during the COVID-19 and post-COVID-19 periods (*p* < 0.05), along with a notable decline in penicillin use. Watch and Reserve antibiotic use rose significantly (*p* < 0.05), while Access group use fell from 67% to 49.2%. **Conclusions:** These findings underscore the lasting impact of the COVID-19 pandemic on antibiotic prescribing patterns and emphasize the urgent need for strengthened antimicrobial stewardship efforts to ensure rational antibiotic use and combat antimicrobial resistance.

## 1. Introduction

Improper use of systemic antibiotics is a major issue in hospitals. Avoiding unnecessary prescribing and ensuring proper administration of antibiotics can contribute to lowering antimicrobial resistance (AMR) and improving treatment outcomes [1]. Inappropriate antibiotic prescribing has been identified in 43.8% of patients and has been linked to increased AMR and adverse drug reactions [2]. The misuse of antibiotics, particularly in the early stages of the COVID-19 pandemic, raised concerns about overuse and the potential spread of multidrug-resistant bacteria [3]. AMR increases the risk of poor treatment outcomes, the transmission of infectious diseases, life-threatening complications, and mortality, making it a significant global public health threat [4]. Therefore, reports on antimicrobial consumption at both local and national levels are crucial. Ensuring the rational use of antibiotics is a fundamental aspect of effective infections management [5]. For this reason, monitoring antibiotic utilization within hospitals and healthcare systems is essential [6], particularly for an antimicrobial stewardship program (ASP) [7].

Hospital admission increases the risk of infections caused by hospital-acquired bacterial strains, as well as the transmission of multidrug-resistant strains, leading to increased antibiotic use, which makes the establishment of effective ASPs in intensive care units (ICUs) particularly challenging [8]. In ICUs, 70% of patients received at least one antibiotic either as prophylaxis or therapy, while 54% were either suspected of having or had a confirmed bacterial infection [9].

The appearance of coronavirus 2 (SARS-CoV-2), first identified in December 2019, has put a significant burden on healthcare systems across the globe. Clinical uncertainty in patients with coronavirus disease (COVID-19), along with the lack of effective antiviral treatments, were the main reasons for the extensive use of antibiotics during the early phase of the pandemic [10]. Studies have shown that more than 70% of hospitalized COVID-19 patients were treated with antibiotics primarily due to fear of bacterial co-infection [11]. However, a thorough data analysis revealed that only about 6% of those patients had microbiologically confirmed bacterial co-infection [12]. During the pandemic, the most frequently used antibiotics were those prescribed for community-acquired pneumonia, such as tetracyclines, macrolides, and cephalosporins [13]. Moreover, the use of carbapenems, categorized by the World Health Organization (WHO) as “Watch” antibiotics group, showed a notable rise [14]. Among critically ill patients, antibiotics from the “Watch” group were the most prescribed [8]. The highest antibiotic utilization rate was observed in COVID-19 patients with severe clinical manifestations, on a global average of 81% [6].

A meta-analysis of seven randomized studies assessing the effect of azithromycin on clinical outcomes in COVID-19 patients found that the drug did not reduce mortality rates or hospital stay duration, providing no benefit for these patients [15]. On the other hand, side effects like QT interval prolongation were noted in patients treated with azithromycin, especially when combined with hydroxychloroquine [16]. In general, antibiotics did not lead to better clinical outcomes for COVID-19 patients. In fact, it may have caused severe adverse reactions to individuals without bacterial infection, compared to those who did not receive antibiotics [17].

Bosnia and Herzegovina, a typical example of a low-resource setting (LRS), had restricted access to new therapeutic options for the treatment of the most severe forms of COVID-19 during the pandemic. The shortcomings of healthcare systems in LRS became highly visible during this time, and our country was no exception. In such circumstances, the irrational use of antibiotics became favored [18,19]. Although several studies have analyzed antibiotic utilization in primary care settings in the Republic of Srpska [20,21,22], one of the two constituent entities of Bosnia and Herzegovina, with an estimated population of approximately 1.4 million, to date, only one study has focused on antibiotic utilization in a general hospital [23]. Given the scarcity of literature on antibiotic consumption in LRS, especially in the context of the COVID-19 pandemic, this study was initiated to address this knowledge gap.

The aim of this study was to analyze the antibiotic utilization in the tertiary teaching hospital over the past decade. The study specifically focused on the trends and quality assessment of annual antibiotic consumption before, during, and after the COVID-19 pandemic.

## 2. Results

**Figure 1:**
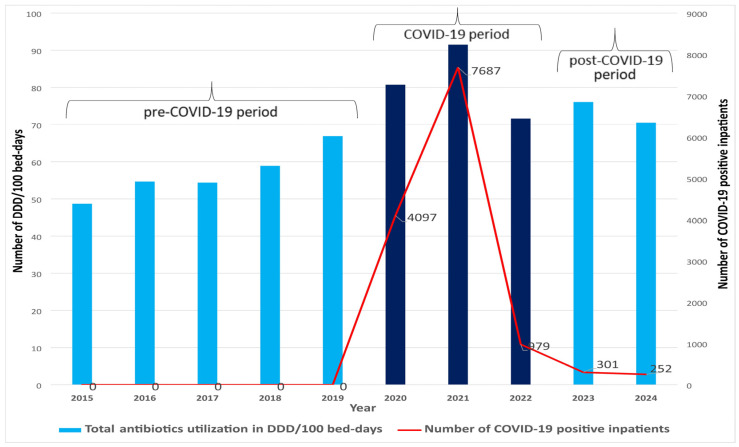
Ten-year trend of antibiotic utilization and number of COVID-19 positive patients at the University Clinical Centre of the Republic of Srpska.

The data presented in Figure 1 indicate a rising pattern of antibiotic consumption during the COVID-19 period, with the highest rate observed in 2021, reaching 91.5 defined daily doses (DDD) per 100 bed-days. Notably, 2021 also marked the peak in the number of hospitalized COVID-19 positive patients (7687).

**Figure 2:**
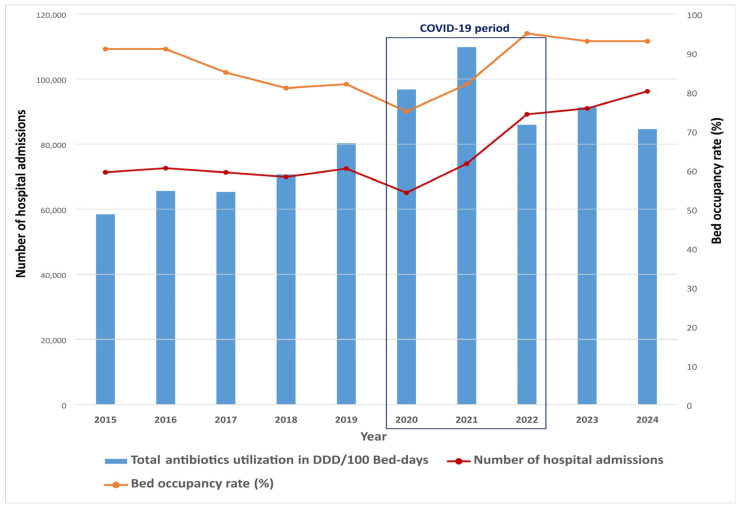
Total antibiotic utilization in relation to hospital admissions and bed occupancy rate.

A reduction in both the number of hospital admissions and bed occupancy rate was observed during the COVID-19 period, while there was a concurrent increase in antibiotic consumption (Figure 2). The highest number of hospital admissions was recorded in the post-COVID-19 period, corresponding with a slight increase in the number of hospital beds and patient days during that time.

The most frequently used antibiotics during the entire study period were cephalosporins (34.5%), penicillin (11.6%), and nitroimidazole-metronidazole (10.5%). The usage of all antibiotic groups during all three study periods is shown in Table 1. A marked increase in the use of carbapenems and other antibiotics was observed during the COVID-19 period compared to the pre-COVID-19 era, with this upward trend continuing into the post-COVID-19 period. A similar pattern was observed in the overall consumption of antibiotics. The use of macrolides, primarily azithromycin, increased more than 2.5 times during the COVID-19 period, particularly in 2020. Although a gradual decline has been noted in the post-COVID-19 period, the azithromycin utilization level has not yet returned to pre-pandemic levels. Furthermore, a decline in penicillin use was evident, despite their status as the most frequently used antibiotic classes.

Compared to the pre-COVID-19 period, the median antibiotic utilization, expressed in DDD per 100 bed-days, showed a significant increase during both the COVID-19 period (80.7 vs. 54.7; *p* < 0.05) and the post-COVID-19 period (73.3 vs. 54.7; *p* < 0.05). Additionally, a comparison of median values among the WHO Access, Watch and Reserve (AWaRe) antibiotic groups revealed a statistically significant rise in the use of both Watch (*p* < 0.05) and Reserve (*p* < 0.05) antibiotics. Statistical analysis across the study periods further indicated a significant increase in the consumption of meropenem (*p* = 0.032), piperacillin/tazobactam (*p* = 0.028), vancomycin (*p* = 0.028), and colistin (*p* = 0.022). A comparison of the median azithromycin use during the COVID-19 period (6.5) versus pre-COVID-19 period (2.2) yielded a borderline significant *p*-value of 0.053. However, the difference in use of the macrolide antibiotic group did not reach statistical significance, largely due to the decline in hospital use of azithromycin during the post-COVID-19 period (Table 2).

The proportion of antibiotics from the Access group used in hospitals decreased from 67% in the pre-COVID-19 period to 49.2% in the post-COVID-19 period. Conversely, the utilization of Watch and Reserve antibiotics increased from 32.6% and 0.4% in the pre-COVID-19 period to 48% and 2.8% in the post-COVID-19 period, respectively. The percentage changes in the use of AWaRe antibiotics across the study periods are presented in Table 3.

## 3. Discussion

The main finding of this study is that a marked increase in antibiotic consumption was observed during the COVID-19 period, with the highest utilization recorded in 2021, the second year of the pandemic, which coincided with the peak in the number of hospitalized COVID-19-positive patients. Moreover, the results clearly showed that the upward trend in antibiotic use was already present before the pandemic and continued in the post-pandemic period, indicating a persistent pattern beyond the direct impact of COVID-19.

Although data from similar studies conducted in LRS are scarce, a multicenter study from Iran, also an LRS, reported an average antibiotic consumption of 121.6 DDD per 100 bed-days during the first six months of the pandemic [24]. Considering that this result emerged from a multicenter analysis where antibiotic use was notably lower compared to our hospital, it may suggest a greater impact of the COVID-19 pandemic on antibiotic consumption in LRS. Contributing factors likely include limited resources, underdeveloped surveillance systems, and the absence of fully established antimicrobial stewardship programs, all of which may have further promoted irrational antibiotic use.

A study conducted in well-developed healthcare systems reported the highest antibiotic consumption at the end of 2020, corresponding to the second wave of the pandemic, although the majority of patients had already received antibiotic therapy during the first wave [25]. Similarly, Henig et al. documented a significantly elevated use of antibiotics during the first wave of the pandemic, particularly between March and mid-April 2020 [10].

During the COVID-19 period, the median antibiotic consumption in our hospital was 80.7 DDD per 100 bed-days, representing a significant increase compared to the pre-pandemic period, where the median usage was 54.7 DDD per 100 bed-days. This finding aligns with the results reported by Abelenda-Alonso et al., who demonstrated that the COVID-19 pandemic led to a substantial rise in overall antibiotic use when compared to 2019 levels [26]. However, contrasting evidence exists; for example, one study analyzing antibiotic consumption from 2015 to 2021 did not observe a statistically significant difference in antibiotic use during the pandemic years (2020–2021) compared to the preceding five-year period [27]. Nonetheless, other reports support our findings, even though they originate from countries with well-developed healthcare systems. A study from Italy, for instance, confirmed increased antibiotic utilization across hospitals during the COVID-19 pandemic [28], further corroborating the widespread impact of the pandemic on antimicrobial prescribing practices.

The rate of antibiotic utilization during the post-COVID-19 period in our study did not return to pre-pandemic levels, although a modest decline was observed compared to the peak during the pandemic. Data on antibiotic consumption trends beyond 2022 remain scarce, particularly in LRS. However, a study by Bellanti et al. [29] reported that antibiotic use in internal medicine wards remained elevated in the post-pandemic period (May to December 2023), with only a slight reduction compared to levels observed during the pandemic. Additionally, the study highlighted that during the COVID-19 pandemic, antibiotic therapy was characterized by prolonged duration and increased costs, both of which persisted even after the pandemic had ended. These findings suggest that the pandemic may have had a lasting impact on prescribing practices and antibiotic stewardship efforts.

The observed reduction in both hospital admissions and bed occupancy rate during the COVID-19 period emphasizes even more the increase in antibiotic consumption during that time. Despite the lower number of hospitalized patients, antibiotic use remained markedly high. In contrast, the post-COVID-19 period was characterized by an increase in hospital admissions, accompanied by a slight rise in bed availability and total patient-days. These dynamics highlight a shift in healthcare system activity and underscore the need for targeted antimicrobial stewardship interventions to address persistently elevated antibiotic utilization beyond the pandemic period.

Given that the risk of AMR escalates with each additional day of antibiotic exposure in both community-acquired and hospital-onset infections [30], it is reasonable to assume that the inappropriate overuse of antibiotics during and after the COVID-19 pandemic may have contributed to shifts in bacterial resistance patterns, as demonstrated in previous studies [31,32]. Furthermore, the increase in hospital admissions observed in the post-pandemic period may have facilitated a rise in healthcare-associated infections and the transmission of multidrug-resistant organisms, thereby driving further antimicrobial consumption [33]. In addition, findings by Calderón-Parra et al. highlighted that patients treated with antibiotics were significantly more likely to experience adverse drug reactions compared to those who did not receive such therapies [2], reinforcing the need for prudent antimicrobial prescribing practices.

Additionally, the Global Antimicrobial Resistance and Use Surveillance System (GLASS) report highlighted that increases in total antibiotic consumption were more commonly observed in low- and middle-income countries [34]. These results should be interpreted with caution, as some countries classified by the World Bank as high-income countries, in terms of healthcare systems, exhibited characteristics of LRS, particularly during the pandemic [35]. One of the key challenges contributing to this trend is the limited availability of cost-effective clinical and biological markers capable of reliably distinguishing between viral and bacterial infections [36]. In such settings, the absence of rapid and accessible diagnostic tools often leads to empirical antibiotic prescribing, which may not always be justified and can further accelerate the development of AMR.

A notable increase in the use of carbapenems, particularly meropenem, as well as vancomycin, piperacillin/tazobactam, and colistin, was observed during the COVID-19 period, followed by a modest decline in the post-COVID-19 period. These findings align with previous reports indicating elevated consumption of carbapenems, piperacillin/tazobactam, and vancomycin in hospital settings during the pandemic [11,28,37]. In addition, a substantial rise in the use of azithromycin was documented during the COVID-19 period compared to other study periods. As anticipated, this pattern mirrors global trends reported in various studies [37,38,39]. The initial endorsement of azithromycin, often in combination with hydroxychloroquine, as a potential therapeutic option for COVID-19 patients likely contributed to its increased use [40]. However, this therapeutic approach was later dismissed due to a lack of clinical efficacy, both as monotherapy and in combination with hydroxychloroquine [41,42]. Furthermore, a meta-analysis of randomized clinical trials highlighted concerns regarding the safety profile of azithromycin, particularly the risk of QT interval prolongation and arrhythmias [15]. Conversely, although the use of piperacillin/tazobactam significantly increased, a downward trend in the total use of penicillins was observed, consistent with findings from other investigations [38,39]. In contrast to our results, Meschiari et al. reported no significant differences in carbapenem and macrolide use during the COVID-19 period (March 2020–November 2021) compared to the pre-pandemic years (January 2015–February 2020) [27].

Our findings revealed notable shifts in antibiotic utilization across the WHO AWaRe categories. Specifically, the use of Watch group antibiotics, such as meropenem, piperacillin/tazobactam and vancomycin, and Reserve group antibiotics, such as colistin, increased significantly during the COVID-19 period. The increased consumption of antibiotics such as colistin is closely linked to the high incidence of intra-hospital infections, particularly those caused by *Acinetobacter baumannii*, which is tightly associated with overcrowding in intensive care units. This issue was especially pronounced in LRS, where the mortality rate for the most severe forms of COVID-19 was also the highest [43,44]. Moreover, the use of Watch group antibiotics continued to rise even in the post-COVID-19 period. In contrast, the proportion of antibiotics from the Access group declined to 49.2% in the post-pandemic period, which deviates from the WHO’s recommendation that at least 60% of total antibiotic consumption should derive from the Access group [45]. During the pre-COVID-19 period, the use of Access antibiotics accounted for 67% of total antibiotic utilization, in alignment with WHO targets; however, this proportion declined by nearly 20% over time. Similar trends were documented in other countries participating in the GLASS surveillance system, where increased use of Watch and Reserve antibiotics was reported [34]. Indeed, several studies have confirmed that Watch antibiotics constituted the most commonly used group during the COVID-19 period [25,39,46]. Supporting this trend, the most recent report from the European Surveillance of Antimicrobial Consumption Network (ESAC-Net) for 2023 found that the median consumption of Reserve antibiotics was significantly higher in 2023 compared to 2019. An overall rise in the use of broad-spectrum antibiotics was also documented [47].

This study has several limitations. Firstly, its retrospective design limited our ability to stratify antibiotic consumption between COVID-19 positive and negative patients, as patient-level diagnostic data were not available for retrospective collection. Given that COVID-19 patients were generally younger and had fewer comorbidities compared to non-COVID-19 patients [8], this may have influenced antibiotic utilization trends across the study periods. Secondly, data on the clinical indication for antibiotic use, duration of therapy, and potential adverse drug reactions could not be retrieved retrospectively, which may have affected the accuracy of DDD estimates—particularly in intensive care settings where dosing strategies can differ. Lastly, as this was a single-center study, the generalizability of the findings to other institutions within the country or region may be limited. Future research should aim to evaluate the association between antibiotic consumption patterns and the development of AMR within the hospital setting.

## 4. Materials and Methods

### 4.1. Study Design and Data Collection

This retrospective observational study was conducted at the University Clinical Centre of the Republic of Srpska (UCC RS), the leading referral healthcare institution in the Republic of Srpska. Annual data of the utilization of antibacterials for systemic use (ATC group J01) from all wards of this university-affiliated, tertiary care teaching hospital were obtained from the Clinical Information System (KIS, Računari d.o.o. Banja Luka, Bosnia and Herzegovina 2011, version 1) for a ten-year period (January 2015 to December 2024). The collected data included the generic name of the antibiotic, ATC code, dosage form, total amount used, and unit of measurement.

The following wards were included in these analysis: Breast Surgery, Medical ICU, Surgical ICU, Otorhinolaryngology, Pediatrics, Pediatric Surgery, Obstetrics and Gynecology, Hematology, Infectious Diseases, Cardiac Surgery, Cardiology, Dermatovenerology, Neurosurgery, Neurology, Oncology, Abdominal Surgery, Orthopedics and Traumatology, Ophthalmology, Plastic and Reconstructive Surgery, Pulmonology, Psychiatry, Thoracic Surgery, Internal Medicine, Urology, Vascular Surgery, Maxillofacial Surgery, and Emergency Medicine.

In the 28-bed medical ICU, approximately 700 patients were hospitalized annually during the pre-COVID-19 period, with an estimated mortality rate of 20%. During the COVID-19 period, this unit exclusively treated COVID-19-positive patients, with the annual number of hospitalizations increasing to approximately 1200 and an estimated mortality rate rising to 50%. In the post-COVID-19 period, both the number of hospitalized patients and the mortality rate returned to pre-pandemic levels. During the pre- and post-COVID-19 periods, around 70% of patients required mechanical ventilation, of which approximately 80% were on invasive mechanical ventilation, and the remainder received noninvasive ventilation. During the COVID-19 period, over 90% of patients required mechanical ventilation.

The study was approved by the Ethics Committee of the UCC RS (approval no. 01-19-120-2/25) and Ethics Committee for human and biological material research of the Faculty of Medicine, University of Banja Luka (approval no. 18/4. 67/25).

### 4.2. Antibiotic Utilization

The total annual consumption of each antibiotic was first converted into grams and then translated into DDD in accordance with the WHO guidelines. All DDDs were calculated using the 2025 version of the ATC/DDD index [48]. To express aggregate utilization, total DDDs were normalized per 100 bed-days [49,50]. A bed-day was defined as one calendar day during which a patient occupied a hospital bed and remained overnight [51]. During the study period, the number of hospital beds gradually increased from 1153 beds between 2015 and 2017 to 1198 beds between 2018 and 2022, and finally to 1289 beds in 2023 and 2024.

Annual antibiotic utilization data over a three year period (2020–2022), defined as the COVID-19 period, were used to assess the impact of the COVID-19 pandemic on antibiotic consumption. The pre-COVID-19 period included the years 2015–2019, while the post-COVID-19 period covered 2023–2024. Antibiotic utilization was compared across these three time periods. Additionally, the number of hospitalized patients over the years was reported, along with the number of confirmed COVID-19-positive patients starting from 2020. In our country, the first confirmed case of COVID-19 occurred in early March 2020 [52].

Since the aforementioned periods are not of equal duration, three two-year intervals were selected to enable a more balanced evaluation of percentage changes in antibiotic utilization: 2015–2016 (pre-COVID-19 period), 2020–2021 (COVID-19 period), and 2023–2024 (post-COVID-19 period) to ensure comparability and minimize bias in trend analysis.

Furthermore, antibiotics were classified into three categories based on the WHO’s AWaRe classification [44] and usage trends within these categories were compared across all years and study periods.

### 4.3. Statistical Analyses

The data were recorded and analyzed using Microsoft^®^ Excel^®^ Version 2013 and IBM^®^ SPSS^®^ Statistics Version 26.0. For continuous variables that did not follow a normal distribution, results were presented as the median number with interquartile range (IQR). Categorical variables were expressed as absolute numbers or percentages. Changes in the utilization rate of antibiotic groups between the study periods were reported as percentages. The Kruskal–Wallis test was used to compare the continuous variables between years and periods. The *p*-value of <0.05 was considered statistically significant, with a 95% confidence interval.

## 5. Conclusions

This study clearly demonstrates a steady increase in antibiotic consumption over ten years, with a peak observed during the COVID-19 pandemic and persistently elevated use in the post-pandemic period. Such a pattern of increased antibiotic use—or misuse—is a well-recognized characteristic of LRS. The rise in broad-spectrum antibiotics from the WHO Watch and Reserve groups is particularly concerning due to its potential to drive AMR. Rational prescribing of antimicrobials is essential to preserve their efficacy and limit resistance. Strengthening antimicrobial stewardship—through local guidelines, appropriate antibiotic selection, timely de-escalation, and regular monitoring—is crucial, particularly in LRS. Future studies should evaluate the long-term impact of pandemic-related antibiotic use and assess the effectiveness of stewardship interventions in these environments.

## Figures and Tables

**Table 1:** Antibiotic group utilization and its changes across study periods (COVID-19 vs. pre-COVID-19 period, post-COVID-19 vs. pre-COVID-19 period and post-COVID-19 vs. COVID-19 period).

	DDD/100 Bed-Days										% Change ^1^		
	2015	2016	2017	2018	2019	2020	2021	2022	2023	2024	COVID vs. Pre- ^2^	Post- vs. Pre- ^2^	Post- vs. COVID ^2^
Cephalosporins	15.6	15.7	18.6	19.9	28.2	23.2	31.4	26.0	27.7	26.3	74.5	72.8	−1.0
Carbapenems	1.2	1.7	1.8	2.5	2.8	5.6	8.4	5.0	5.7	6.1	390.5	313.9	−15.6
Penicillin	8.3	9.9	7.4	9.7	5.3	9.9	8.6	6.3	7.0	6.0	1.6	−28.5	−29.7
Tetracyclines	3.0	3.5	3.2	2.5	2.3	4.4	5.7	1.6	1.7	1.5	55.7	−50.4	−68.1
Quinolones	6.5	6.7	6.1	5.8	5.6	6.7	8.9	6.0	6.7	6.0	18.3	−3.3	−18.2
Aminoglycosides	5.3	5.6	5.5	6.1	6.4	6.6	6.8	6.7	7.2	5.9	23.0	20.1	−2.3
Macrolides	2.0	2.5	2.2	2.8	4.4	9.8	6.6	3.9	2.8	3.8	265.5	47.6	−59.6
Sulfonamides	1.6	1.6	1.4	1.4	1.6	0.9	0.8	1.2	1.4	0.5	−47.7	−39.7	15.3
Lincosamides	0.5	0.4	0.8	0.9	0.4	0.6	1.3	1.1	1.0	0.9	110.5	108.5	−1.0
Nitroimidazole-metronidazole	3.9	6.0	6.0	5.7	8.0	8.2	5.7	8.9	9.9	8.7	40.3	87.5	33.7
Other antibiotics	0.9	1.2	1.3	1.5	1.8	4.9	7.3	5.0	4.9	4.8	485.3	365.9	−20.4
Total	48.7	54.7	54.4	58.9	66.9	80.7	91.5	71.6	76.1	70.5	66.6	41.8	−14.9

^1^ Percentage of change; ^2^ 2015–2016: pre-COVID-19 period, 2020–2021: COVID-19 period, 2023–2024: post-COVID-19 period; Other antibiotics: fosfomycin, chloramphenicol, colistin, linezolid, teicoplanin and vancomycin.

**Table 2:** Changes in overall hospital antibiotic utilization, from 2015 to 2024 (2015–2019: pre-COVID-19 period; 2020–2022: COVID-19 period; 2023–2024: post-COVID-19 period).

	Pre-COVID-19 Period, Median (IQR)	COVID-19 Period, Median (IQR)	Post-COVID-19 Period, Median (IQR)	*p*-Value ^1^
Access	38.5 (3.3)	44.6 (0)	36.1 (0)	0.291
Watch	16.2 (8.2)	32.9 (0)	35.2 (0)	0.032
Reserve	0.2 (0.3)	2.2 (0)	2 (0)	0.032
Total DDD/100 bed-days	54.7 (11.4)	80.7 (0)	73.3 (0)	0.028
Cephalosporins	18.6 (8.5)	26 (0)	27 (0)	0.203
Cefepime	0.3 (0.2)	1.0 (0)	0.6 (0)	0.057
Ceftriaxone	7.7 (6.9)	18.4 (0)	19.3 (0)	0.051
Carbapenems	1.8 (1.2)	5.6 (0)	5.9 (0)	0.032
Meropenem	1.3 (0.9)	4.8 (0)	4.8 (0)	0.032
Penicillin	8.3 (3.4)	8.6 (0)	6.5 (0)	0.422
Amoxicillin	2.5 (1.4)	2.0 (0)	1.9 (0)	0.280
Piperacillin/tazobactam	0.2 (0.2)	0.9 (0)	0.8 (0)	0.028
Tetracyclines	3.0 (0.9)	4.4 (0)	1.6 (0)	0.170
Quinolones	6.1 (0.8)	6.7 (0)	6.4 (0)	0.391
Aminoglycosides	5.6 (0.9)	6.7 (0)	6.5 (0)	0.085
Macrolides	2.5 (1.5)	6.6 (0)	3.3 (0)	0.072
Azithromycin	2.2 (1.5)	6.5 (0)		0.053
Sulfonamides	1.6 (0.2)	0.9 (0)	0.9 (0)	0.055
Lincosamides	0.5 (0.4)	1.1 (0)	0.9 (0)	0.136
Nitrozoimidazole-metronidazole	6.0 (2.2)	8.2 (0)	9.3 (0)	0.088
Other	1.3 (0.6)	5.0 (0)	4.8 (0)	0.028
Vancomycin	1.1 (0.2)	2.9 (0)	2.7 (0)	0.028
Colistin	0.2 (0.3)	1.5 (0)	1.1 (0)	0.022

^1^ Kruskal–Wallis test; IQR—interquartile range.

**Table 3:** World Health Organization AWaRe antibiotic utilization and rate of change across study periods (COVID-19 vs. pre-COVID-19 period, post-COVID-19 vs. pre-COVID-19 period, and post-COVID-19 vs. COVID-19 period).

	N (%)			% Change ^1^		
	Pre-COVID-19 Period (2015–2019)	COVID-19 Period (2020–2022)	Post-COVID-19 Period (2023–2024)	COVID vs. Pre- ^2^	Post- vs. Pre- ^2^	Post- vs. COVID ^2^
Access	190 (67)	130.3 (53.5)	72.1 (49.2)	28.1	−1.6	−23.1
Watch	92.4 (32.6)	106.3 (43.6)	70.4 (48.0)	145.2	135.0	−4.2
Reserve	1.1 (0.4)	7.1 (2.9)	4.1 (2.8)	3356.9	2781.5	−16.6

^1^ Percentage of change; ^2^ 2015–2016: pre-COVID-19 period, 2020–2021: COVID-19 period, 2023–2024: post-COVID-19 period.

## Data Availability

The data presented in this study are available from the corresponding author upon reasonable request.
